# Selfie-Takers Prefer Left Cheeks: Converging Evidence from the (Extended) *selfiecity* Database

**DOI:** 10.3389/fpsyg.2017.01460

**Published:** 2017-09-04

**Authors:** Lev Manovich, Vera Ferrari, Nicola Bruno

**Affiliations:** ^1^The Graduate Center, City University of New York, New York NY, United States; ^2^DiMeC, Neuroscience Unit, Università di Parma Parma, Italy

**Keywords:** *selfiecity*, selfies, self-portraits, left side bias, lateralization

## Abstract

According to previous reports, selfie takers in widely different cultural contexts prefer poses showing the left cheek more than the right cheek. This posing bias may be interpreted as evidence for a right-hemispheric specialization for the expression of facial emotions. However, earlier studies analyzed selfie poses as categorized by human raters, which raises methodological issues in relation to the distinction between frontal and three-quarter poses. Here, we provide converging evidence by analyzing the (extended) *selfiecity* database which includes automatic assessments of head rotation and of emotional expression. We confirm a culture- and sex-independent left-cheek bias and report stronger expression of negative emotions in selfies showing the left cheek. These results are generally consistent with a psychobiological account of a left cheek bias in self-portraits but reveal possible unexpected facts concerning the relation between side bias and lateralization of emotional expression.

## Introduction

Self-portraiture is a well-established genre in the visual arts and it invites scientific scrutiny in many ways. Here, we make a contribution to the study of factors affecting how portraitist arrange their subject in their created image, that is, the problem of composition. Our contribution is different from previous studies of composition for at least two reasons. First, and in contrast with more traditional approaches (see Arnheim, 1954, 1982), we focus on a very specific compositional feature, the choice to display more of the left or right cheek of the subject. Second, and in contrast with traditional studies of portraits and self-portraits (Crozier and Greenhalgh, 1988; Woodall, 1997; Ferrari, 2002; Brilliant, 2004; Calabrese, 2010; Hall, 2014), we study compositional choices using a database of selfies rather than corpora of paintings. Our unusual interests originate from an intriguing bias that has been found to affect posing choices in painted self-portaits as well as in selfies. This bias has potential implications for our understanding of the lateralization of functions in the human brain. Before describing what these implications may be (fourth paragraph of this introduction), we will briefly summarize relevant findings in portraiture and self-portraiture.

Based on studies of art books and catalogs, there is evidence suggesting that artists prefer poses showing left cheeks when composing a portrait, but showing right cheeks when composing their own self-portraits (LaBar, 1973; McManus and Humphrey, 1973; Latto, 1996; Nicholls et al., 1999; Suitner and Maas, 2007; Powell and Schirillo, 2009; Tosun and Vaid, 2014). It has been suggested that both biases may in fact originate from a preference for showing one’s left cheek, as self-portraits are most typically painted while watching oneself in a mirror. The mirror reversal therefore causes the artist to paint an image presenting the right side of the face, but this is in fact the anatomical left side. Supporting this speculation, there is evidence that a right bias in self-portraiture emerged when cheap large mirrors became available (Bruno and Bertamini, 2013) and disappeared when photography became widely available (Lindell, 2012; Bruno and Bertamini, 2013). In addition, evidence supporting a common account for the two biases has recently accrued from studies of selfies. ‘Selfie’ is a generic term referring to photographic self-portraits taken by non-professionals for the purpose of posting on web-based social media. Such casual photographic self-portraits have enjoyed tremendous popularity in the recent years. In addition, because they are taken by everyone and not just by professional artists, selfies are potentially a very rich source of data about compositional choices by individuals with no specific academic training. If such choices are governed by spontaneous preferences rather than academic training or studio conventions, one would expect to see similar biases in selfies and in self-portraits by trained painters. Recent studies have largely confirmed this prediction (Bruno and Bertamini, 2013; Bruno et al., 2014, 2015; Lindell, 2015).

Among these studies, key evidence has been provided by an analysis of the (original) *selfiecity* database containing 3200 selfies posted in *Instagram* from five major world cities (see Tifentale and Manovich, 2014^1^). This database contains two types of selfies: *standard* selfies, which are taken holding a smartphone at arm’s length, and *mirror* selfies, which are taken by photographing a mirror image of onesel, and one’s smartphone, while standing in front of an actual mirror. Interestingly, the analysis revealed a left cheek bias in standard selfies, but a right cheek bias in mirror selfies, independently of city-of-origin or taker sex (Bruno et al., 2015). Given that right cheeks in the photographed mirror images corresponded to the taker’s actual left cheek, this ubiquitous interaction effect is exactly what one would expect if there were a spontaneous, natural preference for displaying the left cheek over the right.

Although quite striking, the results reported by Bruno and collaborators on the *selfiecity* database raise issues. For instance, one obvious question regards the assessments of head rotation. To facilitate comparisons to previous studies, Bruno and collaborators categorized selfies by asking raters to assign each selfie to one of five categories: unambiguously showing more of the left cheek, unambiguously showing more of the right cheek, slightly rotated to the left, slightly rotated to the right, and frontal. The unambiguous categories were determined by looking at the image. If simple observations left some room for doubt, a ruler was used to measure distances from the center of the nose to the visible limit of the cheek and the selfie was categorized as slightly left or right based on which distance was larger. If the difference was smaller than 1 mm, the selfie was categorized as frontal. This method may be criticized in that it leaves some ambiguity on the definition of what is an unambiguous rotation, and in that it collapses moderate with very strong rotations into a single category. A second important question concerns generality: does the bias hold for selfies from other cities (and, therefore, presumably other cultural environments) besides the analyzed five cities (New York City, São Paulo, Berlin, Moscow, and Bangkok). Finally, a third and most important question is what may be the cause of the bias. It has been suggested (Nicholls, 2000; Powell and Schirillo, 2011; Lindell, 2013) that a common cause might be identified in the right-hemispheric specialization for the expression of emotions, which tends to make most of us more expressive on the left side (the right-hemisphere hypothesis; Sackeim et al., 1978; but see also Torro Alves et al., 2008; Prete et al., 2015). If correct, the right hemisphere hypothesis would imply that side biases in self-portraiture have intriguing implications for our understanding of the lateralization of brain functions. It cannot be ruled out, however, that side biases might arise from cultural factors, such as those relating the right and left cheeks to distance in status or gender (Humphrey and McManus, 1973; McManus and Humphrey, 1973; Schirillo, 2000; ten Cate, 2002).

In the current paper we exploited the (extended) *selfiecity* database, now including a sixth city (London, for details see http://selfiecity.net/london/) to address these issues. In particular, we aim at doing two things. First, we want to validate previous conclusions about the existence of a left-side bias in the *selfiecity* database by re-analyzing posing behavior from automatic assessments of head rotation in an extended database now including six rather than five cities. Second, we want to exploit automatic assessments of emotional expression to derive estimates of overall intensity for positive and negative emotions, and to test whether these differ systematically between selfies showing more of the left or the right cheek. This is, to the best of our knowledge, the first attempt to directly relate posing biases in selfies to lateralized emotional expression.

## Materials and Methods

### The (Extended) *selfiecity* Database

To study posing preferences in self-potraits by non-artists, we used the collection of selfies in the (extended) *selfiecity* database. This database consists of 3840 photographic self-portraits spontaneously uploaded on the online photo-sharing social network *Instagram* in six world cities, from December 4 to 12, 2013 (São Paulo, New York City, Berlin, Moscow, or Bangkok) or September 21 to 27, 2015 (London). In addition to the actual images, the database includes a wealth of information about potentially interesting image features, determined by automatic face recognition algorithms (Rekognition by Orbeus, Inc.) and by ratings provided by human observers (Amazon’s Mechanical Turk service). Among these, of interest here are assessments of head rotation around the vertical axis, which measures whether more of the left or right cheek is visible in the self-portrait, estimates of the selfie-taker sex, and estimates of the intensity in expressing a given “mood” (see Emotional Expression Scores), which consists in dimensions that are presumably related to basic emotions.

### Data Validation

Due to imperfections of the automatic face recognition algorithms, the *selfiecity* database contains a few images that are not selfies (see also Bruno et al., 2015). For instance, some of the images in the database contain portraits of more than one individual (“wefies,” see Bruno et al., 2014), or portray an individual who is clearly not holding the camera (which may be consistent with a self-portrait but is not technically a selfie), or contain face-like patterns such as a smiley. Individual inspection of the database led us to identify 269 such images (7% of the total) which were excluded from further analysis.

### Identification of Mirror and Standard Selfies

Although the database includes information about several variables of interest, it did not originally distinguish between standard and mirror selfies. This distinction is critical to test side biases as these two types of selfies make opposite predictions regarding which cheek will be preferred more often depending on whether the preference refers to the actual cheek of the taker or to the position relative to the picture. Selfie type was determined by individually inspecting all images. Selfies were classified as belonging to the mirror type if the camera was visible in the image and it was held by the subject, such that the image was clearly that of the selfie taker photographing his or her own image in the mirror. Otherwise, the image was classified as belonging to the standard type unless there was reason to exclude it as a non-selfie (see Data Validation).

### Identification of Unambiguous Three-Quarter Poses

A certain percentage of selfies consist of frontal poses, which provide no information about biases for one side of the face over the other. Although the percentage of frontal poses is typically not big in selfies, we found that it can vary depending on the nature of the database. It is therefore important to identify frontal poses before comparing percentages of left or right sides. In our previous study (Bruno et al., 2015), we distinguished frontal poses from three-quarter poses by asking raters to classify all images into five categories (unambiguously left, slightly left, frontal, slightly right, unambiguously right) according to the criteria specified in Bruno and Bertamini (2013). Conservatively, we performed all analyses only on the unambiguous poses (about 2400 selfies out of 3200). To identify unambiguous three-quarter poses in the extended *selfiecity* database using continuous head rotation data, we perused several dozens of images and estimated that a reasonable threshold for an unambiguous three-quarter pose can be estimated at ±2.5°. Consistent with the estimate, the percentage of selfies from the updated database entered in the analysis using this threshold proved to be equal to 74%, which is close to the percentage of unambiguous poses from the earlier study (about 78%, see Table 1 in Bruno et al., 2015). We also evaluated the possibility that random error or biases in the automatic recognition algorithm results might impact on how we categorized posing choices. Note that random error would not have such impact as it would merely make the estimates more noisy. Biases would represent a more serious concern but we see no reason that systematic errors, if any, would bias head rotation estimates to the left of right. Moreover, we can exclude that such biases are a serious concern as the automatic estimates turn out to be nicely consistent with our previous estimates based on human ratings (see sections below).

### Emotional Expression Scores

Estimates of the intensity of emotional expression were derived separately for positive and negative emotions according to the following procedure. Based on feature positions estimates from an automatic face recognition software, the *selfiecity* database includes information about “moods,” that is, 0–1 continuous scorings of the intensity of the following face attributes: “surprised,” “happy,” “confused,” “angry,” “sad,” “disgusted,” “calm.” At least one and most often more than one of these scorings are available for each selfie. Given the semantics of these attributes, and after qualitative inspection of corresponding images, we decided to use the score of the “happy” mood as a proxi of the intensity of the expression of a positive emotion, and the average of the “angry,” “sad,” and “disgusted” mood scores as a proxi of the intensity of the expression of a negative emotion. Out of the 3571 selfies that were left in the database after validation, 3202 were assigned a positive emotion score by this procedure and 2707 a negative emotion score. Thus, many selfies, but not all, had some score on both dimensions. These scores were used in further analyses of the association between cheek shown and emotional expression.

## Results and Discussion

### Distribution of the Head Rotation Data

**FIGURE 1:**
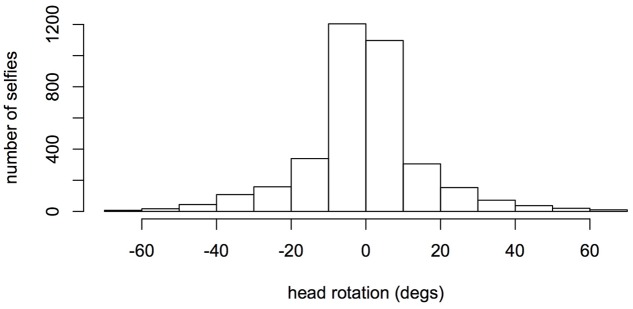
Distribution of head rotation data in the extended *selfiecity* database (six cities). Negative rotations refer to poses showing more the left side of the faces.

**Figure 1** presents the distribution of the head rotation data, as computed by automatic face recognition software (ReKognition by Orbeus, Inc.). The median rotation was – 0.45° and the first and third quantiles of the distribution were -6.645 and 5.435. The minimum and maximum values of the distribution were -69.34 and 68.37. The mean head rotation was -0.64, which is statistically different from zero, *t*(3570) = -2.35, *p* < 0.019. These statistics indicate a slight asymmetry of the distribution due to overrepresentation of negative values, which indeed correspond to head rotations showing more of the left cheek. However, they are of limited interest as the overall distribution conflates standard and mirror selfies. If a bias for showing one’s left cheek exists, we would expect to see an excess of left cheeks in standard selfies, but an excess of right (mirror reflected left) cheeks in mirror selfies. To test this prediction, cheek frequencies need to be compared between selfie types, as we do next.

### Overall Bias for the Left Cheek

**Table 1A** presents the frequency of left- and right-cheek poses (threshold rotation for unambiguous three-quarter view = 2.5°) as a function of selfie type. Given that the right cheek actually corresponds to the reflected left cheek of the taker, the total count of selfies showing the taker’s left cheek is given by the sum of the frequencies of left cheeks in standard selfies and right cheeks in mirror selfies. This adds up to 1417 selfies or 53.8% of the database. This overall bias for showing the left cheek is similar in size to what previously reported for comparable databases (Bruno and Bertamini, 2013; Bruno et al., 2015; Lindell, 2015) and is statistically significant, chi-square(1) = 15.2, *p* < 0.0001, when tested against the null hypothesis that *p*(right cheek) = *p*(left cheek) = 0.5.

**Table 1A:** Frequencies of selfies showing more of the left or right cheek as a function of selfie type.

	Left cheek	Right cheek
Mirror selfies	164	198
Standard selfies	1219	1053


*Classification of cheek shown based on head rotation as estimated by facial recognition software (see text for details). A selfie was classified as displaying a three-quarter view when computed head rotation exceeded 2.5° in one of the two directions.*

### Association between Cheek Shown and Selfie Type

The contingency table in **Table 1A** indeed confirms an excess of right cheeks in mirror selfies, and an excess of left cheeks in standard selfies. A statistical test reveals a small, Cramer’s phi = 0.056, but statistically significant association, chi-square(1) = 8.45, *p* = 0.0037. This is similar to the association (phi = 0.13, *p* < 0.00001) reported by Bruno et al. (2015) who used human raters to detect three-quarter poses and classify cheek preferences in the (original) *selfiecity* database (five cities only).

**FIGURE 2:**
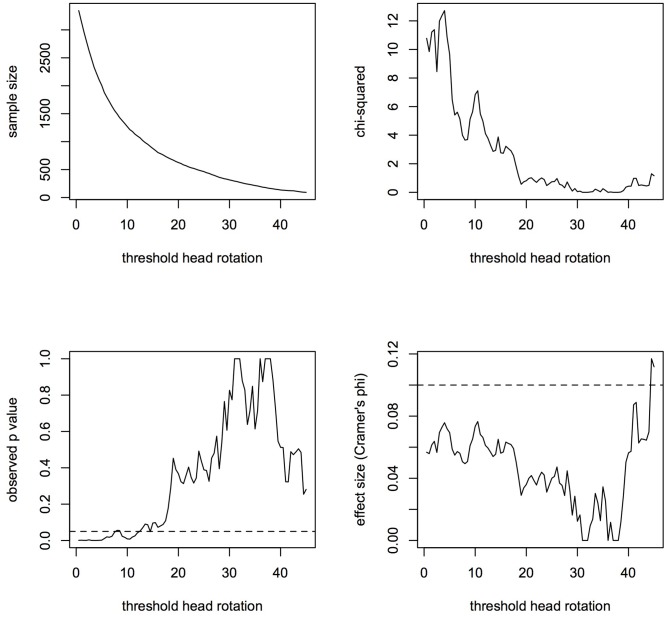
Change in association statistics as a function of threshold head rotation for a three-quarter pose. As thresholds are increased, less and less images are included in the analysis (top, right) and the value of the chi-square statistic reduces (top, left). However, observed *p*-values (bottom, left) remain significant over a range of thresholds well above the value chosen in **Table 1A**.

To make sure that the significant association displayed by **Table 1A** is not just a consequence of choosing a particular threshold value for head rotation, we also studied how association statistics vary with different threshold values, from 0° (equivalent to including all selfies, even if actually consisting of frontal poses) to 45° (including only extreme rotations – almost profile views). **Figure 2** displays the results of this analysis. As one would expect, when the head rotation threshold increases the sample size drops rapidly (top left) as does the value of the chi-square statistic (top right), as less and less images are included in the analysis. However, statistical tests remain significant up to thresholds of about 12°, where the sample size is reduced to only about 1000 images. This patterns suggests that the left cheek bias is robust and does not depend on the choice of a particular value for head rotation as symptomatic of a three-quarter pose.

These results provide two important pieces of information. First, the predicted inversion of the side bias in mirror in comparison to standard selfies is observed even when pose is estimated by automatic recognition software. This suggests that the output of the software provides comparable data to that obtained from human raters (as done in our previous paper, Bruno et al., 2015) and validates the use of the automatic rotation data to predict emotional expression in the selfies, which is the major novel contribution of this paper (see later sections). Second, the predicted inversion remains detectable when the database is expanded to include the sixth city, which adds to the generality of the conclusions.

### Side Bias in Six World Cities

**FIGURE 3:**
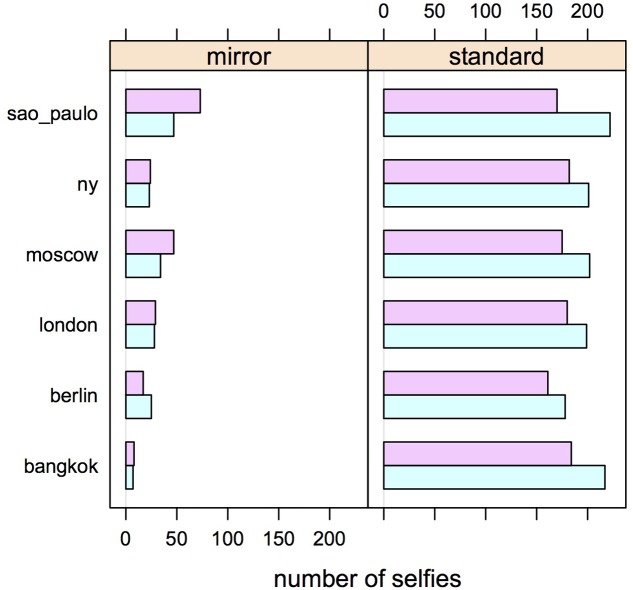
Pose preferences (violet: showing more of the right cheek; pale blue: showing more of the left cheek) in selfies posted on Instagram from six different world cities, as a function of selfie type (mirror or standard).

**Figure 3** presents the frequencies of right or left cheek poses in standard and mirror selfies in each of the six cities. The predicted inversion of the cheek bias is remarkably consistent in different cultural contexts. Indeed, in all six cities we observe an overabundance of left cheeks in standard selfies. In mirror selfies, a corresponding overabundance of right cheeks is observed in five cities. The only exception are the Berlin mirror selfies where we observed 25 left cheeks and 17 right cheeks. Binomial tests based the null hypothesis that in each city 0.4 < *p* < 0.6 of randomly observing the expected inversion yields *p*-values in the range 0.02–0.004, suggesting that this pattern is highly unlikely to be due to chance.

### Side Bias in Females and Males

**FIGURE 4:**
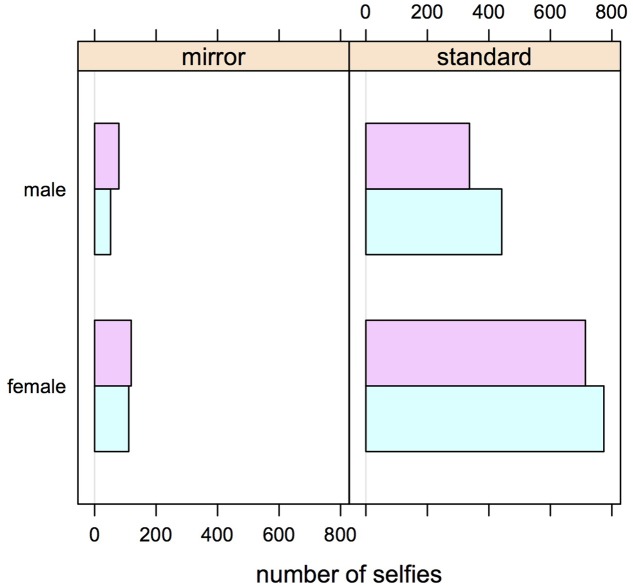
Pose preferences (conventions as in **Figure 3**) in selfies as a function of selfie taker sex.

**Figure 4** present the frequencies of right or left cheek poses, in standard and mirror selfies, separately for females and males. The two subsamples are not completely comparable as women are much more likely to appear in the database than males. Nonetheless, the qualitative pattern of the association between selfie type and cheek remains visible in both sex categories. Separate tests yielded a statistically significant association in the female, chi-square(1) = 7.4, *p* < 0.007, but not in the male subset, chi-square(1) = 1.1, *p* = 0.3. These tests may be taken as indication that the side bias inversion is not present, or is not as general, in male as compared to female selfies. This conclusion, however will need further verification from other databases of images as in previous work (not including mirror selfies, Bruno and Bertamini, 2013) we found similar biases in males and females. We suggest that more data are needed here, especially given that both male and mirror selfies are underrepresented on Instagram relative to females and standard selfies.

### Analysis of Emotional Expression Scores

**FIGURE 5:**
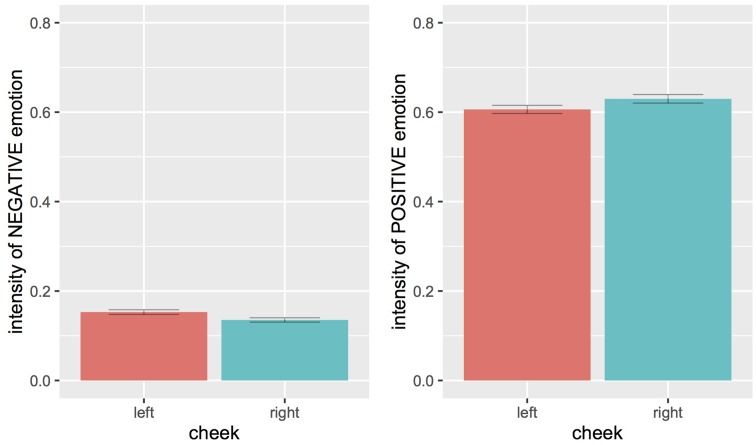
Main effects of cheek shown on the intensity in expressing negative (left) or positive (right) emotions. Each bar represents the group mean intensity, and each corresponding error bar represents one standard error of the mean.

**FIGURE 6:**
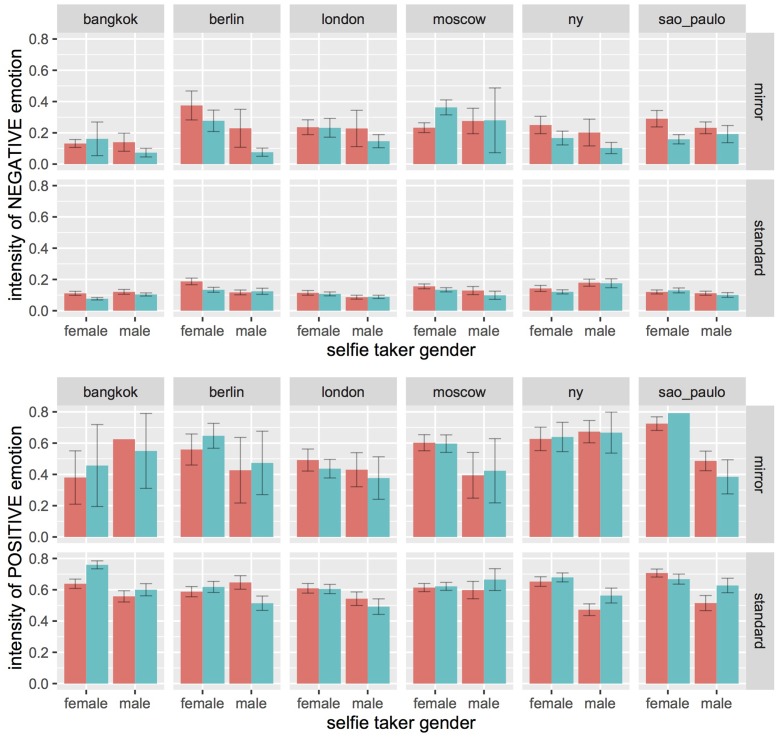
Selfie-taker gender, selfie type, city of origin, and cheek shown (red = left; pale blue = right, as in **Figure 5**) as predictors of the intensity in the expression of negative (top) or positive (bottom) emotions. Each bar represents the group mean intensity, and each corresponding error bar represents one standard error of the mean.

**Figure 5** presents the average intensities of negative and positive emotion scores in selfies showing a left- or right-cheek bias. **Figure 6** presents the same data as a function of all the variables considered here, namely, the cheek bias, the selfie-taker gender (male or female), the type of selfie (mirror or standard), and the city of origin. We performed two separate ANOVAs for the positive and negative emotion data. The dependent variable was the intensity of the expressed emotion, and the independent variables were the cheek shown (left or right), the selfie-taker gender (male or female), the type of selfie (mirror or standard), and the city of origin. These two ANOVAs were performed separately as the positive and negative scores had diametrically opposed distributions (right-tailed for negative, skewness = 2.16; but left-tailed for positive, skewness = -0.33). The results of these two ANOVAs are described in detail in the next sections.

### Effect of Valence

Overall, positive emotions were expressed more strongly than negative emotions. We take this as self evident after visual inspection of **Figure 6**, as every single bar in the bottom row is higher than the corresponding bar in the top row.

### Effect of Cheek Shown

Negative emotions were expressed more strongly in selfies showing the left cheek; positive emotions, conversely, were expressed more strongly in selfies showing the right cheek (**Figure 5**, see also **Table 1B**). In the negative emotions scores, the difference was statistically significant, *F*(1,1979) = 5.26, *p* = 0.022. In the positive emotions scores, however, the difference failed to reach statistical significance, *F*(1,2325) = 2.85, *p* = 0.092.

**Table 1B:** Mean intensity (SEM) of estimated positive and negative emotional expression for selfies showing more of the taker’s left or right cheek (frontal poses not included).

	Negative	Positive
Left cheek	0.154 (0.005)	0.606 (0.009)
Right cheek	0.136 (0.005)	0.629 (0.01)


### Effect of Type of Selfie

Negative emotions were expressed more strongly in mirror selfies; positive emotions, conversely, were expressed more strongly in standard selfies (see **Table 2**). In the negative emotions scores, the difference was statistically significant, *F*(1,1979) = 109, *p* < 0.0000001. In the positive emotions scores, however, the difference failed to reach statistical significance, *F*(1,2325) = 3.8, *p* = 0.051.

**Table 2:** Mean intensity (SEM) of estimated positive and negative emotional expression for two kinds of selfie (standard or mirror).

	Negative	Positive
Mirror	0.239 (0.013)	0.584 (0.018)
Standard	0.127 (0.003)	0.622 (0.007)


### Effect of Taker Gender

Both positive and negative emotions were expressed more strongly by females (see **Table 3**). In the positive emotions scores, the difference was statistically significant, *F*(1,2325) = 38.2, *p* < 0.0000001. In the negative emotions scores, conversely, the difference failed to reach statistical significance, *F*(1,1979) = 2.24, *p* = 0.135.

**Table 3:** Mean intensity (SEM) of estimated positive and negative emotional expression in selfies by takers classified as men or women.

	Negative	Positive
Female	0.149 (0.005)	0.645 (0.008)
Male	0.137 (0.006)	0.551 (0.012)


### City of Origin

The intensity of both negative and positive emotions changed as a function of the city of origin for the selfies (see **Table 4**), *F*(5,1979) = 5.26, *p* < 0.0001 and *F*(15,2325) = 4.3, *p* = 0.0007. In the negative emotion scores, Tukey HSD *post hoc* pairwise comparisons identified significant differences between Bangkok and Berlin, *p* = 0.002, Moscow, *p* = 0.004, and New York, *p* = 0.032; as well as between London and Berlin, *p* = 0.024, and London and Moscow, *p* = 0.043. In the positive emotion scores, they identified significant differences between London and Bangkok, *p* = 0.012, and London and São Paulo, *p* = 0.002.

**Table 4:** Mean intensity (SEM) of estimated positive and negative emotional expression in selfies posted from six world cities.

	Negative	Positive
Bangkok	0.102 (0.006)	0.649 (0.016)
Berlin	0.168 (0.011)	0.595 (0.018)
London	0.123 (0.008)	0.563 (0.017)
Moscow	0.170 (0.009)	0.611 (0.015)
New York	0.153 (0.009)	0.618 (0.016)
São Paulo	0.147 (0.008)	0.654 (0.015)


### Two-Way Interactions: Cheek Shown by Selfie Type, Gender, or City

Overall differences in intensity of emotional expression between left and right cheek selfies were similar in standard or mirror selfies, male or female takers, and in each of the different cities (see **Figure 2**). Accordingly, the relevant two-way interactions yielded *F* values < 1 in both the positive and the negative emotion scores.

### Three-Way Interactions: Cheek Shown by Gender and City

There was no evidence of a three-way interaction between cheek shown, gender and city of origin. Statistical tests yielded F < 1 and *F*(1,2325) = 1.525, *p* = 0.18 and in the negative and positive emotion scores, respectively.

### Three-Way Interactions: Cheek Shown by Selfie Type and Gender

There was no evidence of a three-way interaction between cheek shown, selfie type and gender. Statistical tests yielded *F*(1,1979) = 2.8, *p* = 0.095 and F < 1 in the negative and positive emotion scores, respectively.

### Three-Way Interactions: Cheek Shown by Selfie Type and City

In the negative emotion scores, the analysis provided evidence of a three-way interaction between cheek shown, selfie type and city, *F*(5,1979) = 3.21, *p* = 0.007. Inspecting the interaction means indicated that this effect was due to a different pattern of the Moscow selfies in comparison to the other five cities. In all the other five cities, the pattern of the three-way interaction was consistent with the main effect of cheek shown in both standard and mirror selfies. Said otherwise, all the means were consistent with higher emotional expression on the left cheek. In the Moscow selfies, however, this difference was visible in the standard selfies, but not in the mirror selfies that revealed a large difference in favor of the right, not left cheek. In the positive emotion scores, conversely, there was no evidence of a three-way interaction, F < 1.

### Four-Way Interactions: Cheek Shown by Gender, Selfie Type, and City

There was no evidence of a four-way interaction between the four independent variables. In the negative emotion scores, the statistical test yielded *F*(5,1979) = 1.2, *p* = 0.3; in the positive emotion scores, it yielded F < 1.

#### Summary of Main Findings

We have performed a novel analysis of side biases in selfies using the (extended) *selfiecity* database that contains 1000s of photos from six global cities. We used continuous measures of head rotation, and estimates of the intensity of emotional expression provided by computer vision analysis of the photos.

Our results confirm the finding of previous studies: selfie-takers have a bias toward showing more often the left instead than the right cheek. This bias is present regardless of the city where the photos were taken and of the gender of the takers. The only qualitative exception to the predicted pattern was found in the Berlin mirror selfies, which failed to show the expected bias for right cheeks (which, in the mirror reflection, corresponded to left cheeks of takers). However, given the relatively small number of mirror selfies in comparison to standard selfies, after dividing the sample in smaller subsets random fluctuations are to be expected and cannot be taken as evidence of systematic differences. Overall, our findings are therefore quite consistent with earlier reports, including our own which used a different method for assessing the side preference, in supporting a left-side bias independent of sociocultural factors. Especially, interesting in this respect is that cultural differences did emerge from our analysis. For instance, mirror selfies seem to be more prevalent in certain cities than in others. Despite these differences, however, a side bias remained apparent.

Concerning the role of emotional expression on the face as a potential factor in determining the side bias, our analysis revealed several interesting effects. In particular, our findings support the conclusions that negative, but not positive emotions are expressed more strongly in mirror than in standard selfies; that females express positive, but not negative, emotions more strongly than males; that there are cultural differences in emotional expression as shown by differences between some cities and others. These effects were in part already described by Tifentale and Manovich (2014) and may be regarded as at least partly consistent with common opinions about selfies. For instance, it has often been noted that selfie taking and posting is much more a female than a male behavior, and this may reflect in better skills at positive self presentation by females. A quick perusal of the *selfiecity* online database demonstrates that aggressive or provocative posing is often present in mirror selfies by both males and females. Although the reason for this phenomenon is unclear, it may underlie the bias in favor of negative emotions. Finally, styles of social interaction in South America and especially Asia generally predict that during self-presentation individuals might tend to prefer friendly expressions and especially to inhibit unfriendly expressions, in comparisons to North America or Northern Europe. Interestingly London, in comparison to the other five cities, yielded the lowest average intensity of positive emotional expression but also a very low average intensity of negative emotional expression (almost as low as Bangkok, which however has the highest intensity of positive expression), a finding that is surprisingly consistent with the a commonly held stereotype that social interactions in Great Britain favor restraint on emotional expression of all kinds.

Although the present study was not aimed at investigating sociocultural determinants of emotional expressions in selfies, we believe these observations are interesting and generally in support of the conclusion that the *selfiecity* database is representative of selfie taking behaviors across different cultural contexts. Importantly, these main effects seem to be essentially independent of potential sociocultural moderators, as shown by the absence of any two-way or higher-order interaction. The only exception was the cheek by city and selfie type three-way interaction, which however seems to be driven only by some peculiarity of self presentations in a mirror by Moscow selfie takers. However, given that mirror selfies were much less frequent than standard selfies, it is difficult to decide whether this effect truly reflects a cultural modulation of cheek preferences or merely statistical variation.

Critically for the aims of the current paper, our results provide evidence for a difference in emotional expression between the left and the right cheek, but only for negative emotions. Indeed we observe that, at least for negative emotional expressions, selfies showing the left cheek tend to express the emotion more strongly than selfies showing the right cheek A similar, but in the opposite direction, difference was observed for positive emotions, which tended to be expressed more strongly on the right side. This difference however failed to reach significance.

Our aim in assessing the intensity of emotional expression was to explore whether differences in emotion intensity can be observed depending on the posing bias for the left or right side of the face. Overall our results provide mixed evidence in support of this hypothesis. Although we did find that emotions were expressed more strongly in selfies showing the left side of the face, this observation was limited to negative emotions. In contrast, our results with positive emotions did not allow us to draw a clear-cut conclusion. On one hand, positive emotions yielded a trend in the opposite direction in comparison with negative ones. If this were indeed the case, the results could be interpreted as supporting the so-called *valence* hypothesis on the expression of facial emotions (Davidson et al., 1987; Bourne, 2010). According to this hypothesis, the right hemisphere specializes more for the expression of negative emotions, whereas the left hemisphere specializes more for the expression of positive emotions. On the other hand, this trend failed to reach significance. We cannot exclude, therefore, that there was in fact no difference in positive emotional expression between the right and the left cheek.

Either way, these findings may be interpreted as evidence that if the lateralization of emotional expression plays a role in the left side bias, this has to do more with the expression of negative than positive emotions. This possibility is unexpected and, to the best of our knowledge, has never been advanced before. In the context of the current study, however, we suggest it should remain a speculation in need of further support. The main reason for this note of caution is the nature of our emotional expression scores, which were not derived from psychometrically validated assessments of the intensity of expressed emotions but from machine-based estimates of “moods” within a commercial facial recognition algorithm. We cannot therefore be 100% certain that our scores were pure valid measures of emotional expression, although they are likely to reflect it at least to some degree. Because the *selfiecity* mood data were originally obtained from a commercial site, we have been unable to obtain information on the proprietary algorithm that was used to derive the original mood scores. A natural solution to this problem will be to run the image database through a scoring procedure by human raters using psychometrically validated scales.

## Conclusion

The present results provide converging evidence for a natural, culture-independent preference to display the left cheek. It has been suggested (Nicholls et al., 1999; Powell and Schirillo, 2011; Lindell, 2013) that the basis for this preference might be identified in the right-hemispheric specialization for the expression of emotions, which tends to make most of us more expressive on the left side (Sackeim et al., 1978; Harris and Lindell, 2011; Blackburn and Schirillo, 2012; but see also Torro Alves et al., 2008; Prete et al., 2015). Evidence that a robust left cheek preference can be observed in casual self-portraits created by individuals that are unlikely to have had much exposure to academic training in the arts is certainly consistent with this proposal. Further study will be needed to determine whether the bias can be linked specifically to hemispheric asymmetries in emotional expression. As a first step in this direction, here we have shown that, at least for negative emotions, selfies showing the left cheek of the taker tend to have higher intensities of emotional expression. Further research is needed to determine if this conclusion is general and can form part of an explanation for the left side posing bias.

## Ethics Statement

The data were derived from publicly available photographic self-portraits. These were posted by individuals that, in so doing, implicitly gave permission to anyone to view and use the photographs as stated in the Instagram privacy policy (https://www.instagram.com/about/legal/privacy/). Thus Ethics approval was not needed as also stipulated in our earlier paper (Bruno et al., 2015; see http://journals.plos.org/plosone/article?id=10.1371/journal.pone.0124999). In addition, and to avoid any copyright issues, we did not reproduce any of the photograph in the paper or elsewhere, and were never aware of the identity of the individuals who posted the selfies.

## Author Contributions

LM: data collection and analysis, critical revision of the article; VF: data collection and analysis; NB: conception or design of the work, data analysis, drafting the article.

## Conflict of Interest Statement

The authors declare that the research was conducted in the absence of any commercial or financial relationships that could be construed as a potential conflict of interest.

## Footnotes

1 http://selfiecity.net
